# Frequency-Tunable Terahertz Plasmonic Structure Based on the Solid Immersed Method for Sensing

**DOI:** 10.3390/s21041419

**Published:** 2021-02-18

**Authors:** Toshio Sugaya, Yukio Kawano

**Affiliations:** 1 Department of Electrical and Electronic Engineering, School of Engineering, Tokyo Institute of Technology, Tokyo 1528552, Japan; sugaya.t.ac@m.titech.ac.jp; 2 Laboratory for Future Interdisciplinary Research of Science and Technology, Tokyo Institute of Technology, Tokyo 1528552, Japan

**Keywords:** terahertz, plasmonic, high transmission, near-field, sub-wavelength

## Abstract

Terahertz waves are located in the frequency band between radio waves and light, and they are being considered for various applications as a light source. Generally, the use of light requires focusing; however, when a terahertz wave is irradiated onto a small detector or a small measurement sample, its wavelength, which is much longer than that of visible light, causes problems. The diffraction limit may make it impossible to focus the terahertz light down to the desired range by using common lenses. The Bull’s Eye structure, which is a plasmonic structure, is a promising tool for focusing the terahertz light beyond the diffraction limit and into the sub-wavelength region. By utilizing the surface plasmon propagation, the electric field intensity and transmission coefficient can be enhanced. In this study, we improved the electric field intensity and light focusing in a small region by adapting the solid immersion method (SIM) from our previous study, which had a frequency-tunable nonconcentric Bull’s Eye structure. Through electromagnetic field analysis, the electric field intensity was confirmed to be approximately 20 times higher than that of the case without the SIM, and the transmission measurements confirmed that the transmission through an aperture had a gap of 1/20 that of the wavelength. This fabricated device can be used in imaging and sensing applications because of the close contact between the transmission aperture and the measurement sample.

## 1. Introduction

The terahertz (THz) frequency band has a substance-specific absorption spectrum (fingerprint spectrum) for various chemicals, macromolecules and water vapor. Furthermore, its energy is much lower than that of X-rays, and, therefore, it is being considered for non-destructive inspections, particularly in the biotechnology and medical fields. This is because of its ability to reduce the damage it can cause to the measurement sample [1,2,3]. In addition, the wavelengths of THz waves are approximately 3 mm (0.1 THz) to 30 µm (10 THz), which is tens or hundreds of times longer than those of visible light (400–800 nm). They can be problematic due to the limitation called the “diffraction limit”. For instance, when THz waves are applied in cancer cell detection, the size of a single cell is approximately 10 µm, which is very small when compared with the wavelength of THz waves, making it difficult to detect a single cell by focusing light with a common lens. Additionally, wavelength is often a bottleneck in the measurement of minute amounts of drugs, rare substances and complex tissue-intertwined sites. Furthermore, light focusing is crucial for handling light and is beneficial not only for the imaging and sensing of small objects, but also to highly miniaturize and sensitize detectors. To achieve successful light focusing beyond the diffraction limit, antennas [4,5,6,7] and metamaterials [8,9] have been proposed and confirmed to be effective tools. In this study, we focused on one of the plasmonic structures, the Bull’s Eye (BE) structure [10,11,12]. The BE structure consists of concave–convex concentric circles, with a transmission aperture at its center. When the structure is irradiated by a THz wave, the surface plasmon (SP) is excited and propagated to the transmission aperture, which is where the propagated SP is enhanced, allowing for sufficient light to be focused even when the transmission aperture is sub-wavelength. The strong light focusing characteristic and transmission enhancement of the BE structures can be utilized in various applications, such as imaging, sensing and enhancing the sensitivity of detectors [13,14,15,16]. A common challenge for these focusing devices that overcome the diffraction limit is that the structure-derived resonant frequency of the device, once fabricated, cannot be changed. To tackle this challenge, we proposed that a nonconcentric BE (NCBE) structure [17] is one of the possible solutions.

In this study, by applying the solid immersion method (SIM) to the proposed NCBE structure, the focusing region was reduced to approximately 1/20 of the wavelength and the electric field intensity and transmission coefficient were further enhanced. In addition, through sample measurements using the NCBE, even for samples in which no significant difference was observed in far-field measurements, a large signal change was confirmed by the local field enhancement of this structure. We believe that this study will significantly contribute to various applications of THz waves.

## 2. Materials and Methods

The SIM utilizes the effect of the effective wavelength being shortened to 1/n by the refractive index n in the dielectric, and it is generally used in a lens [18,19]; additionally, its adaptation to the BE structures is also being considered for application in thin-film sensing [20]. We applied this method to the NCBE structure with a high-resistance Si (n: 3.43), which facilitates high transmission in the THz band. By using this effect, miniaturization of the device, enhancement of the electric field and an improvement of the transmission coefficient due to the electric field confinement effect of the dielectric materials was expected. First, we used electromagnetic field analysis to design the device structure and evaluated the electric field intensity and the transmission coefficient. Subsequently, we fabricated the device and evaluated the transmission by measurements using THz Time-Domain Spectroscopy (THz-TDS). 

### 2.1. Device Design Using Electromagnetic Field Analysis

**Figure 1:**
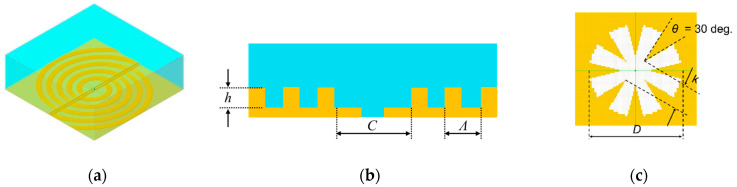
Electromagnetic analysis model. (**a**) Whole model. Blue and yellow parts represent the Si substrate and Al, respectively, (**b**) Schematic view of the cross section and (**c**) Enlarged view of the central aperture.

The Poynting software, developed by Fujitsu, was employed for electromagnetic analysis to design and evaluate the desired device. Furthermore, the software uses the finite difference time domain (FTDT) method. Figure 1 shows the analytical model. The number of concave–convex concentric circles was set to 6 to shorten the analysis time, and the Si substrate was in contact with the outer perfect matched layer wall to simulate infinite thickness. The metal was Al, and the Lorentz–Drude model was used for Si and Al. The design frequency was set from 1.0 THz to 1.5 THz, and the following equation was used for the curve design calculations [17];

(1) $$r=\frac{a}{4}\cdot \frac{c}{\left(f_{2}-f_{1}\right)\;\cdot \;\frac{\pi }{\varphi }+f_{1}},\;0\;<\;\varphi <\;\pi ,\;\alpha \;=\;5,7,9,11\ldots$$

Frequencies *f*_1_ and *f*_2_ were used at *n* times the target frequency to account for the shortening of the effective wavelength by the SIM. Table 1 shows the device structure parameters of the designed NCBE with the SIM and those of the conventional NCBE without the SIM. By using the SIM, it is possible to reduce the size of the device to approximately 1/11 of its area when fabricating the structures with the same number of convex concentric circles. In actual measurements, the number of concave–convex concentric circles for the SIM increases when compared with the conventional NCBE because the THz-irradiated area is fixed by the measurement system, and more SPs are expected to be excited in this case. However, in this analysis, the irradiated area and the analysis space were miniaturized to the values calculated by the refractive index in order to make a comparison with the same number of concave and convex concentric circles.

### 2.2. Device Fabrication

**Figure 2:**
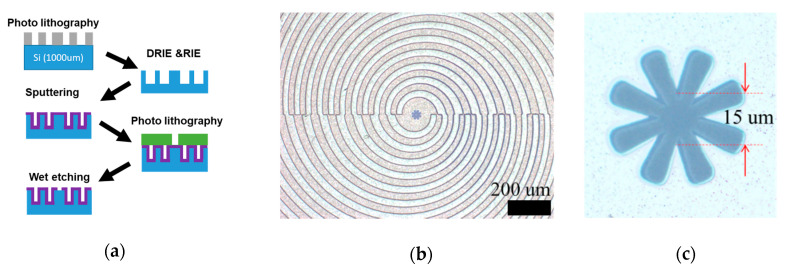
(**a**) Fabrication process overview, (**b**) Overhead optical image (the transmission aperture is located at the center) and (**c**) Magnified optical image of the transmission aperture. The diameter of the narrowest part is approximately 75 µm in the conventional nonconcentric Bull’s Eye (NCBE) [14], whereas it is 15 µm in this device.

Figure 2 shows the device fabrication process and the fabricated devices. High-resistance Si (10,000 Ω/cm) with a thickness of 1 mm was used as a substrate. The concave–convex was fabricated by deep reactive ion etching, with a resist mask made by photolithography. Subsequently, a 500-nm Al film was sputtered to cover the sidewall surface. Finally, wet etching of the Al film with a resist mask, which was made by photolithography, was used to create the transmission aperture. The number of concave–convex concentric circles was 50 because the beam diameter of the measurement system was approximately 5 mm. The transmission area of the fabricated device, where the narrowest distance of the aperture was the diameter, was approximately 1/10 of that of the conventional NCBE structure.

## 3. Results

### 3.1. Electromagnetic Field Analysis Result

**Figure 3:**
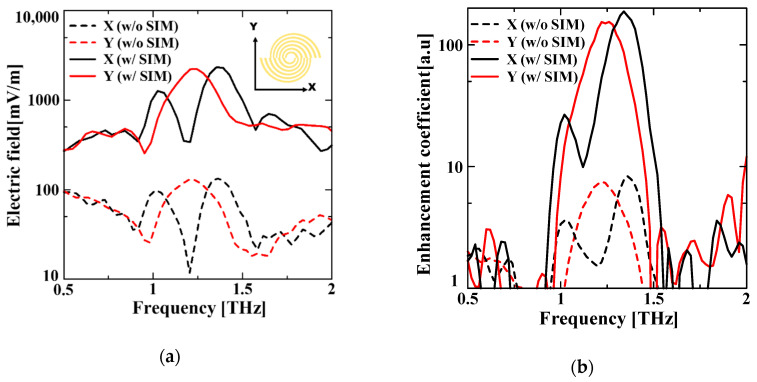

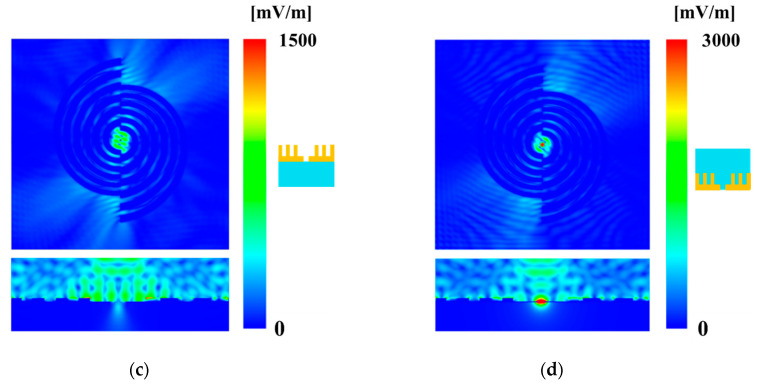
Results of the electromagnetic field analysis. (**a**) Maximum electric field intensity spectrum at the center of the transmission aperture. (**b**) EC spectrum transmitted through a 5 µm square area located in the center of the transmission aperture. X and Y indicate the direction of excitation of the incident pulse in the orthogonal direction. (**c**) Electric field distribution of a conventional NCBE structure. (**d**) Electric field distribution of the SIM NCBE structure. The corresponding frequencies are 1.36 THz and 1.34 THz of the peak frequency, respectively. The overhead view is the lower surface of the concave part of the structure, and the cross-sectional view shows the electric field distribution in the section parallel to the direction of the excitation through the center of the structure.

Figure 3 presents the results of the electromagnetic field analysis. Figure 3a shows the maximum field intensity spectrum at the center of the transmission aperture, where X and Y are the two types of orthogonal polarization of the excitation pulse. By applying the SIM, we achieved a 20 times improvement in the maximum electric field intensity and reduced the device area size by approximately 11 times, with the same number of concave–convex concentric circles. Figure 3b depicts the enhancement coefficient (EC) spectrum, which is defined as the energy transmitted through a 5 µm square area, located in the center of the transmission aperture and normalized by the energy transmitted through the same area when the plane wave is irradiated in free space. Using the SIM increased the EC by approximately 7 to 9 times when compared with that obtained without the SIM. This result indicates that the energy can be focused in a smaller space. Furthermore, there is a possibility of downsizing the transmission aperture and enhancing the transmitted light intensity by employing the SIM. The results of the analyzed spectral peaks are summarized in Table 2. Figure 3c,d show the electric field intensity distributions at the peak frequency for the two cases of with and without the SIM. Both figures show that the excited SPs were concentrated around the central transmission aperture; however, the field intensity of the case with the SIM was distributed in the center more strongly. The cross-sectional views show that the shape of the electric field distribution after transmission is very different for the two cases. In the conventional model, the radiations were observed, whilst they were localized in the proposed method with the SIM. The distribution pattern of the conventional model can be explained by its radiating side, which has a dielectric (Si) as a support substrate. In addition, the radiating side of the proposed method with the SIM is an air layer, which confines the electric field inside the dielectric.

### 3.2. Transmission Measurement Results

**Figure 4:**
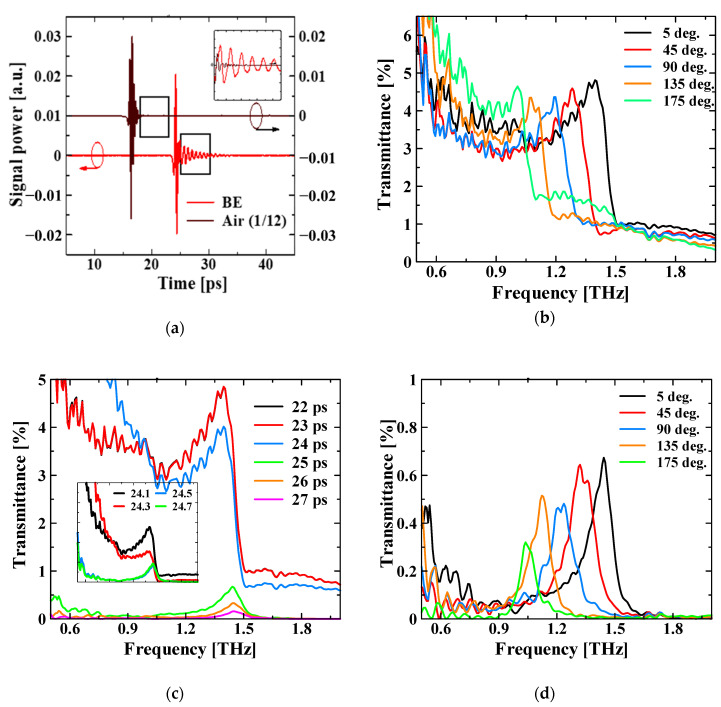
Results of THz-TDS transmission measurements. (**a**) Time waveforms. The intensity for air was reduced to 1/12 of the time domain waveform of the NCBE structure. The inset figure compares the two signals after adjusting their rise times, and the BE time signal contains many delay components. (**b**) FT transmission spectrum of the time waveform. Changing the incident polarization angle shifts the peak frequency. (**c**) Spectrum for a limited time window of the FT. The times listed are the start times of the time window and the end time is unified at 45 ps. (**d**) Transmission spectra when the time window is set from 25 ps to 45 ps.

The fabricated device was evaluated using transmission measurements with a THz-TDS system (TAS-7500 Advantest. Co., Tokyo, Japan). Figure 4 shows the measured time domain signal and transmission spectra. In the enlarged part in Figure 4a, a large delay is observed in the time domain waveform of the NCBE structure when compared with that of air. It is generally known that the propagation velocity of the SP is lower than that of light [21,22], which confirms the SP excitations. Figure 4b is a Fourier-transformed (FT) transmission spectrum of the time waveform, where the angle represents the polarization of the irradiated THz pulse to the device. We confirmed that the resonant frequency can be tuned by changing the incident polarization. The peak frequency varied from 1.01 THz to 1.40 THz, which is 32% of the band, with a center frequency of 1.21 THz. A supplement to the measurements is given in Appendix A.

Figure 4c shows the transmission spectra of the time waveform with different time windows in the FT. By extracting and transforming only the delayed component of the time waveform, it is possible to separate the irradiated and transmitted THz pulse from the excited SP component. As a result, there was only a slight change in the peak frequency; however, a clear peak can be seen in the spectral shape, which also confirms that the SP was excited. By changing the start time of the time window, the start time of the SP component was approximately 24.5 ps (see the bottom left of Figure 4c). Figure 4d shows the polarization dependence of the time window, starting at 25 ps. The results of the spectral peaks in Figure 4b and d are summarized in Table 3.

**Figure 5:**
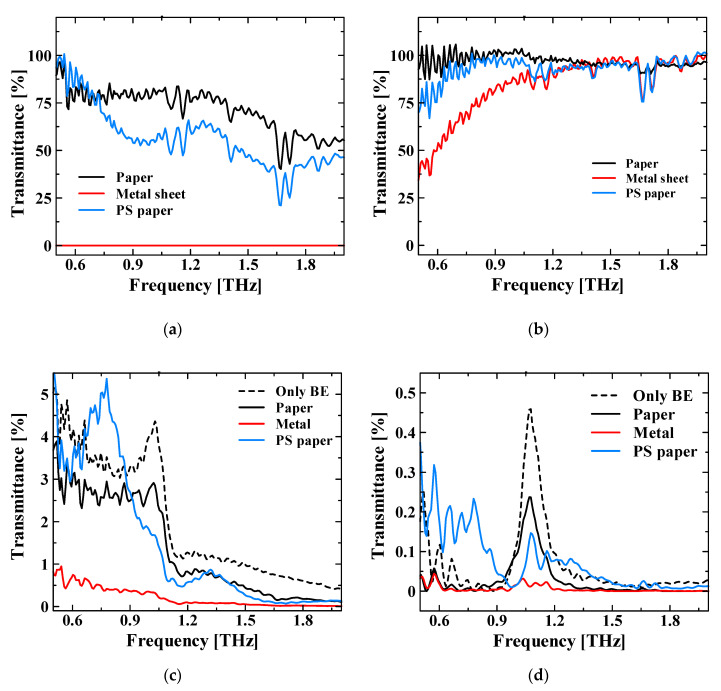
Results of the sample transmission measurements. (**a**) Transmission spectra of the samples that cover the entire optical path (approximately 5 mm in diameter). (**b**) Transmission spectra of the samples with 1 mm width and 2 cm length. (**c**) Transmission measurement results of the samples in (**b**) using the NCBE structure. Different spectra were measured for each sample, confirming that the NCBE structure functions as a lens. (**d**) Transmission spectra for different time windows. For each time window, the transmission time calculated from the refractive index and thickness was added to the start time of the time window for the NCBE window alone.

Subsequently, we examined the effect of the local enhanced field in the near-field region by the NCBE structure on the sensing measurements, when compared to the far-field measurements without the NCBE. Furthermore, the measurement samples for the transmission measurements were paper, metal and photosensitive paper. Figure 5a depicts the transmission spectra of a sample that completely covers the optical path of the THz-TDS system. No transmission was observed for metals; however, specific spectra were observed for paper and photosensitive paper. Figure 5b shows the transmission spectra of the samples of 1 mm width at the center of the optical path, which is narrower than the approximately 5 mm focal width of the THz-TDS system. The measured spectrum varied slightly depending on the sample placement, and, as an overall tendency, it has a high transmittance and loses its characteristic spectrum. Figure 5c shows the transmission spectra when the NCBE was used as a lens. Samples were placed onto the NCBE and in full contact with it. The transmission spectra of the samples, excluding the photosensitive paper, proves the influence of the NCBE’s original spectrum; however, there is a difference in the peak frequency transmittance for each sample. The peak frequency of the photosensitive paper changed, which is a notable measurement result.

## 4. Discussion and Future Plan

The results of the electromagnetic field analysis confirm that the electric field intensity was significantly enhanced and that the SP was localized at the center of the transmission aperture by using the SIM. Additionally, this device structure allows for direct contact between the transmission aperture and sample. These two features provide a remarkable advantage in sensing and imaging. Furthermore, the localization of the transmitted wave means that only small signals can be detected when the measurement system performs far-field measurements. These results suggest that the device can be used more effectively as a type of probe, rather than as a lens or filter in transmission measurements. Additionally, if a small detector is installed near the transmission aperture for coupling, this problem is solved and it is considered a reasonable candidate as a tool for enhancing detection sensitivity and selecting detection frequency. Applying the SIM to the NCBE structure allows for electric field intensity enhancement, miniaturization of the device and superior design flexibility. In particular, when sufficient electric field intensity and transmission coefficients are available, the design frequency range of tuning can be further extended and the size of the transmission aperture can be further reduced. When the frequency range is extended by structural changes, the change in curvature may cause a reduction in the Q value. Therefore, this drawback should be carefully considered when used in spectroscopy. The FT techniques, such as the one used in this study, and the methods that use only a transmission aperture in the metal plane for reference [23], are difficult to use for samples with low transmission in order to improve the Q value of SPs. Additionally, methods that vary the time window need to consider each sample’s physical property and size. Consequently, it is desirable to use it as a probe, as described earlier in this paper. Another possible application of this method of changing the transmission spectrum is to change the refractive index of the dielectric material itself. For instance, refractive indices of phase-change materials can be tunable. Furthermore, VO_2_ has been studied in the THz region [24,25,26]. As the BE structure requires constant transmission, it is necessary to employ a material to ensure sufficient transmittance and to avoid rapid changes in the refractive index and in transmission (conductivity) by strict control measures [27].

Although the sample measurement in this study was made simply by placing small samples on the NCBE structure, because of a problem in the experimental system, we succeeded in measuring the transmission of THz waves through a sub-wavelength aperture. Consequently, we confirmed the advantage of using the SIM to improve the light focus in the transmission region. In the small sample measurement using NCBE, different spectra were measured for each sample, which was not achieved in the normal far-field measurement and, therefore, confirms its function as a lens. However, many areas for improvement still exist. Regarding the transmission of paper, although the use of the NCBE was successful in sensing small samples, a large difference existed between the bulk transmission rate of 87% and the NCBE transmission rate of 66% (full time window) and 52% (limited time window). Furthermore, the metal transmission was 7.6–2.4%, which is slightly anomalous considering that it is completely shielded by metal. These phenomena could have been because of insufficient contact or changes in the optical path that were caused by the sample. Nevertheless, our future study will focus on improving the measurement system to fully exploit the performance of the NCBE for various applications.

**Figure 6:**
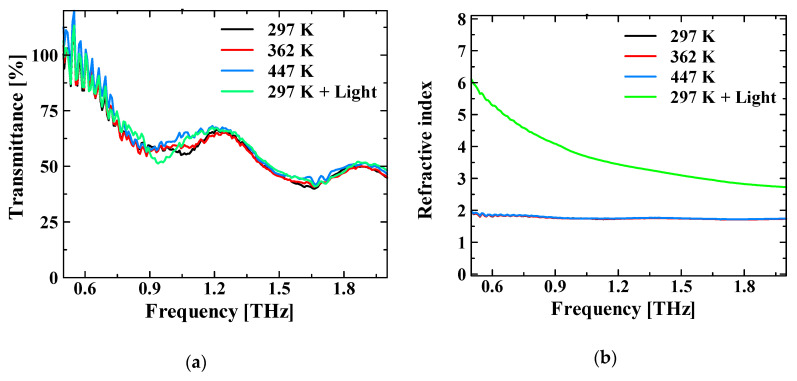
Results of the photosensitive paper measurements. (**a**) Transmission spectra and (**b**) Refractive index spectra. Neither the transmittance nor the refractive index changes when the temperature is changed; however, the refractive index changes significantly when the paper is irradiated with pulsed light.

A very strong focus of THz waves could be the reason for the thermally reversible change in the peak frequency in the transmission measurement of the photosensitive paper. In other words, it absorbs heat and changes its color from black to green or white, and then returns to its original color after some time. This color change may indicate a change in the material, which may create a special transmission spectrum. To investigate this phenomenon, two measurements were performed: (1) measurement of the spectrum of the photosensitive paper heated by a heater; and (2) the measurement of the spectrum while irradiating it with a visible-light pulse laser. Figure 6 shows the transmittance and refractive index spectra for each case. External changes did not affect the transmittance. The refractive index remained constant when the temperature changed; however, it changed significantly when the photosensitive paper was irradiated with the laser pulses. In conclusion, the refractive index changes when the photosensitive paper is irradiated with time-varying electromagnetic waves in a sufficiently strong electric field.

Another factor is that the THz wave, transmitted through the NCBE structure, contains a Z component (a direction parallel to the irradiated wave) in addition to the excitation field component (XY) of the irradiated pulse; it originates from scattering at the transmission aperture. Therefore, the influence of the electric field component, which is not usually irradiated in transmission measurements of THz-TDS, should be examined. Consequently, liquid crystals can be reasonable candidates for use as photosensitive paper. The actual material of the photosensitive paper used in this measurement was unknown because it was a commercial product. However, it is known that liquid crystals are typically used as photosensitive papers; therefore, the material used in our future measurements could be liquid crystal.

In the future, we would like to profoundly investigate the SP excitation and its transmission in the NCBE structure by fully examining the measurement system and sample selection.

## 5. Conclusions

In this study, we proposed the application of the SIM to the NCBE structure. The electromagnetic field analysis confirmed that this method improves the electric field intensity by 20 times and the enhancement factor by 7–9 times compared with those of the previous NCBE structure. In addition, it is useful for focusing light in the sub-wavelength region. The transmission measurements on the fabricated device proved that we succeeded in detecting signals passing through a transmission sub-wavelength aperture, with a gap of approximately 1/20 of the wavelength, and in tuning the resonance frequency from 1.44 THz to 1.04 THz by changing the incident polarization. In the sample-based measurements, the NCBE was used as a lens to confirm minute differences in the spectrum of each sample that cannot be confirmed by the usual far-field measurement system. We believe that this study improves the sub-wavelength transmission performance of the NCBE structure and contributes prominently to THz applications such as improving the flexibility in the design of structures; it also suggests the possibility of coupling with small detectors to improve performance and the possibility of using it for probes. 

## Figures and Tables

**Table 1:** Device structure parameters (unit: µm).

Solid Immersed	*Λ*	*C*	*h*	*D*	*k*
W/O	300–200	750–500	30	150	75
W	88–60	220–150	9	40	15

**Table 2:** Analyzed spectral peaks.

Solid Immersed	X Excitation			Y Excitation		
	Peak Frequency (THz)	|E| Peak (mV/m)	EC Peak (a.u.)	Peak Frequency (THz)	|E| Peak (mV/m)	EC Peak (a.u.)
W/O	1.36	133	8.33	1.22	128	7.48
W	1.34	2328	188	1.22	2227	153

**Table 3:** Measured spectrum peaks.

Polarization	Full Time Window		25–45 ps Time Window	
	Peak (THz)	Transmission (%)	Peak (THz)	Transmission (%)
0 deg.	1.40	4.81	1.44	0.67
45 deg.	1.28	4.59	1.32	0.64
90 deg.	1.19	4.36	1.24	0.48
135 deg.	1.08	4.35	1.12	0.51
180 deg.	1.01	4.64	1.04	0.32

**Figure A1:**
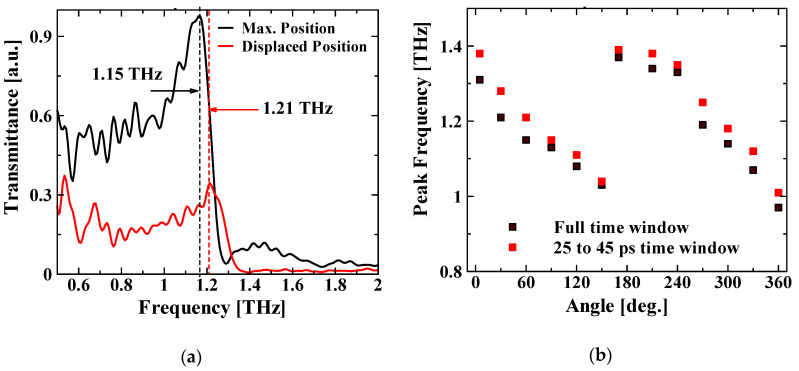
Polarization dependence measurements of the transmission spectrum. (**a**) Variation in the transmission spectrum when the position in the optical path is changed by approximately 3 mm at the same polarization angle and (**b**) Resonance frequency changes when polarization is changed at intervals of 30° from 0° to 360°. 0°, 180°, and 360° are measured as 5°, 175°, and 355°, respectively, as the semicircular shape makes spectral measurement difficult.

## Data Availability

Data sharing not applicable.

## Appendix A

This section describes the details of the transmission measurement for polarization dependence in Section 3.2. The polarization of the THz-TDS is fixed and the relative polarization angle between the device and the incident light is changed by rotating the platform on which the device is mounted.

Because THz-TDS light is invisible, the device is moved to the position where the transmitted signal intensity is the strongest within the area considered to be the optical path. This alignment has a significant effect on the transmitted signal. Figure A1a shows the change in the transmission spectrum when the device position is changed by approximately 3 mm, with the polarization angle fixed. It can be observed that the transmittance changes significantly, but the resonant frequency is not substantially affected.

Figure A1b shows the dependence of the resonant frequency on the polarization angle when the polarization angle is changed by 360°. Two periodic frequency shifts occur, which are consistent with the periodic (every 180°) structure of the NCBE.

## References

[1] Ashworth P.C., MacPherson E., Provenzano E., Pinder S.E., Purushotham A.D., Pepper M., Wallace V.P. (2009). Terahertz pulsed spectroscopy of freshly excised human breast cancer. Opt. Express.

[2] Reid C.B., Reese G., Gibson A.P., Wallace V.P. (2013). Terahertz time-domain spectroscopy of human blood. IEEE J. Biomed. Health Inform..

[3] Wang J., Stantchev R.I., Sun Q., Chiu T., Ahuja A.T., MacPherson E.P. (2018). THz in vivo measurement: The effects of pressure on skin reflectivity. Biomed. Opt. Express.

[4] Llombart N., Chattopadhyay G., Skalare A., Mehdi I. (2011). Novel Terahertz Antenna Based on a Silicon Lens Fed by Leaky Wave Enhanced Waveguide. IEEE Trans. Antennas Propag..

[5] Arikawa T., Morimoto S., Tanaka K. (2017). Focusing light with orbital angular momentum by circular array antenna. Opt. Express.

[6] Okamoto O., Fujimura N., Crespi L., Kodera T., Kawano Y. (2019). Terahertz detection with an antenna-coupled highly-doped silicon quantum dot. Sci. Rep..

[7] Jakhar A., Dhyani V., Das S. (2020). Room temperature terahertz detector based on single silicon nanowire junction-less transistor with high detectivity. Semicond. Sci. Technol..

[8] Kannegulla A., Cheng L.J. (2016). Subwavelength focusing of terahertz waves in silicon hyperbolic metammaterials. Opt. Lett..

[9] Salama N.A., Desouky M., Obayya S.S.A., Swillam A. (2020). Free space super focusing using all dielectric hyperbolic metamaterial. Sci. Rep..

[10] Ishihara K., Hatakoshi G., Ikari T., Minamide H., Ito H., Ohashi K. (2005). Terahertz Wave Enhanced Transmission through a single Subwavelength Aperture with Periodic Surface Structures. Jpn. J. Appl. Phys..

[11] Drezet A., Genet C., Ebbesen T.W. (2008). Miniature Plasmonic Wave Plates. Phys. Rev. Lett..

[12] Beruete M., Beaskoetxea U., Zehar M., Agrawal A., Liu S., Blary K., Chahadih A., Han X., Navarro-Cia M., Salinas D.E. (2013). Terahertz Corugated and Bull’s-Eye Antennas. IEEE Trans. Terahertz Sci. Technol..

[13] Heggie T.J., Naylor D.A., Gom B.G., Bordatchev E., Trimboli M.G. (2016). Enhanced Terahertz Transmission Through Bullseye Plasmonics Lenses Fabricated Using Micromilling Techniques. Plasmonics.

[14] Ishihara K., Ikari T., Minamide H., Shikata J., Ohashi K., Yokoyama H., Ito H. (2005). Terahertz Near-Field Imaging Using Enhanced Transmission through a Single Subwavelength Aperture. Jpn. J. Appl. Phys..

[15] Deng X., Kawano Y. (2018). Surface plasmon polariton graphene midinfrared photodetector with multifrequency resonance. J. Nanophotonics.

[16] Liu S., Shou X., Nahata A. (2011). Coherent Detection of Multiband Terahertz Radiation Using a Surface Plasmon-Polariton Based Photoconductive Antenna. IEEE Trans. Terahertz Sci. Technol..

[17] Deng X., Li L., Enomoto M., Kawano Y. (2019). Continuously Frequency-Tunable Plasmonic Structure for Terahertz Bio-sensing and Spectroscopy. Sci. Rep..

[18] Fletcher D.A., Crozier K.B., Guarini K.W., Minne S.C., Kino G.S., Quate C.F., Goodson K.E. (2001). Microfabricated silicon immersion lens. J. Microelectromech. Syst..

[19] Mansfield S.M., Kino G.S. (1990). Solid immersion microscope. Appl. Phys. Lett..

[20] Hailu D.M., Alqarni S., Cui B., Saeedkia D. Terahertz Surface Plasmon Resonance Sensor and Bull’s eye Structure for Material Sensing. Proceedings of the 38th International Conference on Infrared, Millimeter, and Terahertz Waves (IRMMW-THz).

[21] Temnov V.V., Woggon U., Dintinger J., Devaux E., Ebbesen T.W. (2007). Surface plasmon interferometry: Measuring group velocity of surface plasmons. Opt. Lett..

[22] Wang K., Mittleman D.M. (2006). Dispersion of Surface Plasmon Polaritons on Metal Wires in the Terahertz Frequency Range. Phys. Rev. Lett..

[23] Iguchi T., Sugaya T., Kawano Y. (2017). Silicon-immersed terahertz plasmonic structures. Appl. Phys. Lett..

[24] Wen Q., Zhang H., Yang Q., Xie Y., Chen K., Liu Y. (2010). Terahertz metamaterials with VO_2_ cut-wires for thermal tunability. Appl. Phys. Lett..

[25] Li L., Zhang Y., Li J., Yang Y., Huang J., Ma C., Ma Z., Zhang Z., Liang L., Yao J. (2020). Frequency-switchable VO_2_-based coding metasurfaces at the terahertz band. Opt. Commun..

[26] Zhong M. (2020). Design and verification of a temperature-sensitive broadband metamaterial absorber based on VO2 film. Opt. Mater..

[27] Karaoglan-Bebek G., Hoque M.N.F., Holtz M., Fan Z., Bernussi A.A. (2014). Continuous tuning of W-doped VO_2_ optical properties for terahertz analog applications. Appl. Phys. Lett..

